# Elucidation of Grassy Off-Odor Formation in Wuyi Rock Tea Processed from Dew-Soaked Leaves via GC–MS, Molecular Docking, and Sensory Evaluation

**DOI:** 10.3390/foods15162894

**Published:** 2026-08-19

**Authors:** Chengjiang Wei, Weiwei Wu, Zhechen Han, Peng Zhang, Chenglong Li, Siqing Du, Qi Lin, Fuming Lin, Weijiang Sun

**Affiliations:** 1 College of Horticulture, Fujian Agriculture and Forestry University, Fuzhou 350002, China; wcj4649@163.com (C.W.); wweiwei0601@163.com (W.W.); hzc9986@163.com (Z.H.); pengz6564@gmail.com (P.Z.); 52403030013@fafu.edu.cn (C.L.); dusiqing@foxmail.com (S.D.); lq1005344693@163.com (Q.L.); 2 Anxi College of Tea Science, Fujian Agriculture and Forestry University, Quanzhou 362400, China

**Keywords:** Wuyi rock tea, dew-soaked leaves, GC–MS, grassy off-odor, molecular docking, aroma addition test

## Abstract

Wuyi rock tea (WRT) processed from dew-soaked leaves is often characterized by a pronounced grassy off-odor defect. However, the key odor-active compounds and their potential underlying mechanisms remain unclear. In this study, quantitative descriptive sensory analysis, GC–MS, GC–O, molecular docking, and aroma addition tests were used to systematically elucidate the chemical basis and potential perceptual mechanisms of the key grassy off-odor in WRT processed from dew-soaked leaves. The results showed that 16 volatile compounds were significantly associated with dew, among which (E,E)-2,4-heptadienal, octanal, hexanal, and heptanal were identified as the major odor-active compounds responsible for the grassy off-odor. The accumulation of these aldehydes may be associated with lipid peroxidation under the influence of dew. Aroma addition and omission tests confirmed that (E,E)-2,4-heptadienal contributed significantly to grassy off-odor formation and that additive effects existed among the four compounds. Molecular docking predicted that the four key odor-active compounds interacted with human olfactory receptors, including OR1A1 and OR10J5, through hydrogen bonds and hydrophobic interactions, thereby affecting grassy off-odor perception, with predicted binding energies below −4.7 kcal/mol. This study elucidated the effects of dew on the formation of the characteristic aroma of WRT and provides a scientific basis for tea quality regulation and processing improvement.

## 1. Introduction

Wuyi rock tea (WRT), one of China’s ten most renowned teas, is distinguished by its unique quality attributes and profound cultural heritage. As a representative oolong tea of northern Fujian, WRT is highly appreciated for its mellow taste and rich aroma, particularly its characteristic “rock flavor and floral aroma” profile [1]. Aroma is considered one of the most important indicators for evaluating WRT quality, and its formation is jointly determined by the quality of fresh leaves and processing practices. Fresh leaves provide the material basis for tea quality, whereas processing technologies govern the transformation and stabilization of characteristic aroma compounds. To date, extensive research has focused on the influence of processing conditions on aroma formation. However, despite being a critical factor affecting fresh leaf quality, the role of dew in shaping the characteristic aroma profile and overall quality of WRT has not been systematically elucidated [2,3]. This knowledge gap has, to some extent, hindered advances in process optimization and quality regulation of WRT.

The distinctive climatic conditions of the Wuyi Mountain region contribute substantially to the unique quality characteristics of WRT [4]. However, during the production season, prolonged rainfall frequently increases ambient humidity, resulting in the condensation of atmospheric moisture and the formation of dew on leaf surfaces during the early morning hours [5]. Fresh tea leaves bearing visible dew at harvest are commonly referred to as dew-soaked leaves. Traditional tea-processing practices generally suggest that dew-soaked leaves are unsuitable for the production of high-quality WRT, as they not only increase the difficulty of harvesting and processing but may also adversely affect the quality of the final product. Luo et al. reported that the contents of aroma compounds in WRT manufactured from leaves harvested under high-humidity conditions or on rainy days decreased by 40–70% compared with those harvested on sunny days, accompanied by a pronounced grassy off-odor characteristic [6]. These findings indicate that moisture adhering to leaf surfaces can substantially affect the development of tea aroma quality. However, the key odor-active compounds directly responsible for the grassy off-odor, their sensory contributions, and their potential interactions with olfactory receptors remain unclear.

In recent years, elucidating the formation mechanisms of undesirable flavor compounds in tea has become one of the major challenges in tea chemistry research. Chen et al. reported that alcohols, such as linalool and geraniol, were positively associated with tea quality, whereas aldehydes, including hexanal and (E,E)-2,4-heptadienal, exhibited significant negative correlations with tea quality [7]. Zheng et al. further demonstrated that grassy off-odor compounds, such as (Z)-2-hexenal and (E,E)-2,4-heptadienal, can be generated from polyunsaturated fatty acids through LOX/COX-mediated lipid oxidation pathways, highlighting the pivotal role of lipid oxidation in tea aroma formation [8]. Based on these findings, it is reasonable to speculate that dew deposition may alter lipid metabolism during tea processing, thereby promoting the accumulation of volatile aldehydes associated with grassy off-odor characteristics and ultimately leading to the pronounced grassy off-odor observed in WRT manufactured from dew-soaked leaves. However, the chemical basis underlying grassy off-odor formation in WRT processed from dew-soaked leaves remains insufficiently understood. In particular, investigations into the molecular interactions between key grassy off-odor compounds and olfactory receptors are still lacking.

Quantitative descriptive analysis (QDA) has been demonstrated to be a reliable sensory analysis method for quantifying taste and aroma intensities by training panelists with standardized reference descriptors and calibrating their ratings on a linear scale. When combined with aroma addition and omission tests, QDA enables comparison of the effects and relative contributions of key differential compounds to the sensory quality of foods [9,10]. Molecular docking is a computational simulation technique used to predict interactions between molecules and target receptors, such as proteins. Docking results are evaluated based on binding affinity (docking score) and interaction types, such as hydrogen bonds and hydrophobic interactions [11]. Molecular docking has been applied to investigate the formation mechanism of the characteristic honey-like aroma in honey-scented black tea by exploring the interactions between olfactory receptors and characteristic aroma compounds [12]. The application of molecular docking to tea aroma research represents a novel strategy for identifying key aroma compounds [13,14].

In this context, the present study investigated WRT manufactured from simulated dew-soaked and dew-free leaves. An integrated analytical approach combining QDA, gas chromatography–mass spectrometry (GC–MS), gas chromatography–olfactometry (GC–O), molecular docking, and aroma addition/omission experiments was employed to systematically identify the aroma compounds associated with grassy off-odor and to elucidate their underlying mechanisms. The specific objectives of this study were as follows: (1) to characterize the aroma profile of WRT processed from simulated dew-soaked leaves using GC–MS, GC–O, and QDA, to systematically identify the key aroma-active compounds, and to construct an aroma flavor wheel; (2) to elucidate, at the molecular level, the binding modes between grassy off-odor compounds and human olfactory receptors through molecular docking analysis; and (3) to further confirm the major compounds responsible for grassy off-odor perception through aroma addition and omission experiments. This study provides important scientific evidence and technical support for quality control and process optimization of tea manufactured from dew-soaked raw materials.

## 2. Materials and Methods

### 2.1. Chemical Reagents

A standard mixture of n-alkanes (C7–C40) was purchased from O2SI (Shanghai, China). Dichloromethane (chromatographic grade) was obtained from Mingrui Co., Ltd. (Shenzhen, China). Ethyl caprate (chromatographic grade) was purchased from Sigma-Aldrich (Shanghai, China). Authentic standards of hexanal (99%), heptanal (99%), octanal (98%), and (E,E)-2,4-heptadienal (98%) were obtained from Aladdin (Shanghai, China).

### 2.2. Preparation of WRT Samples Processed from Dew-Soaked and Dew-Free Leaves

**Figure 1:**
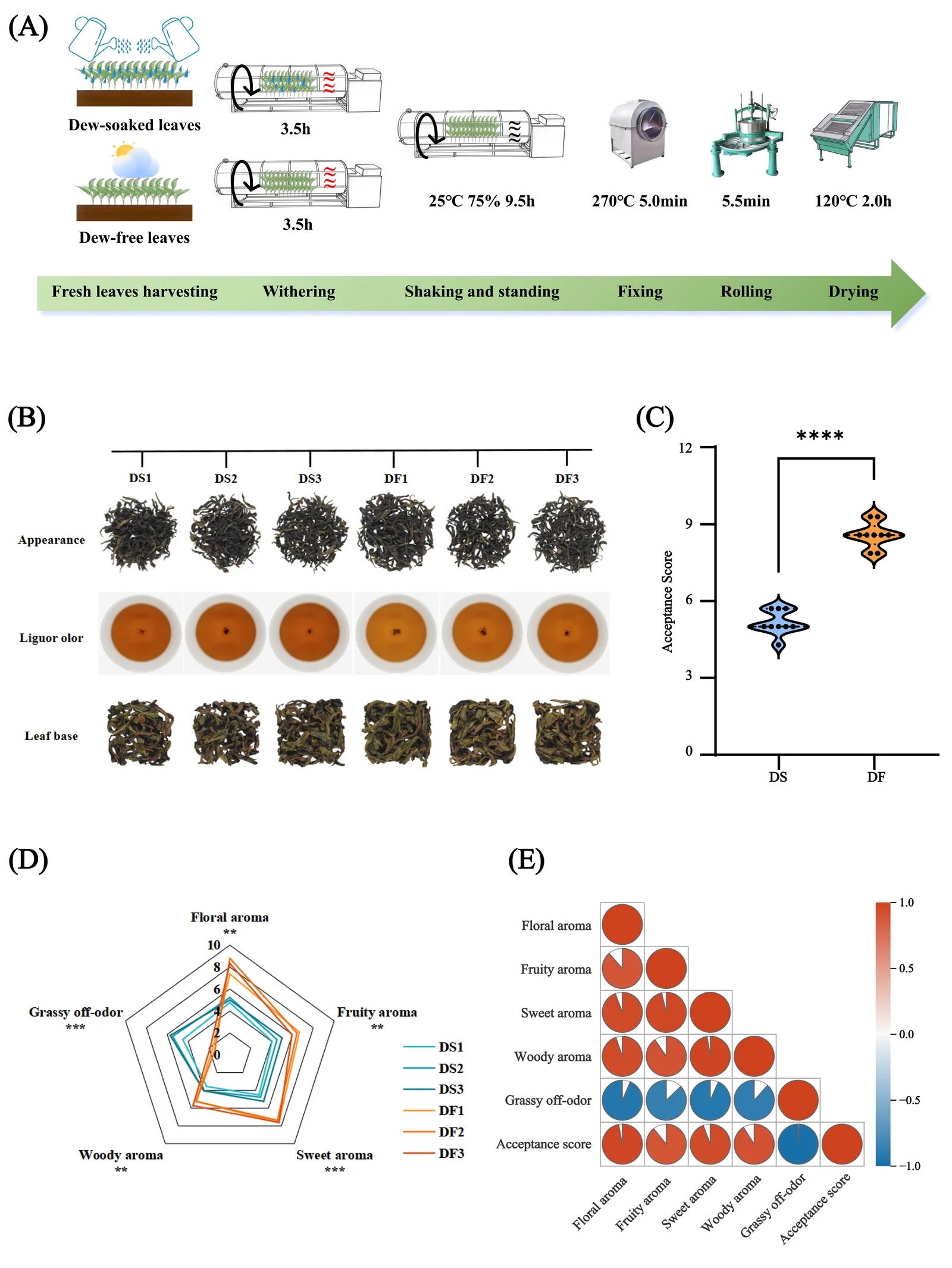
(**A**) The manufacturing process of primary Wuyi rock tea (WRT). (**B**) Images of dry tea, liquor color, and infused leaves. DS1–DS3 and DF1–DF3 represent three independent biological replicates of WRT processed from dew-free leaves and simulated dew-soaked leaves, respectively. (**C**) Violin plot of WRT aroma acceptance score. (**D**) Quantitative descriptive analysis results of WRT. (**E**) Correlation between aroma attributes and acceptance; Blue and orange indicate positive and negative correlations, respectively. ** indicates *p* < 0.01, *** indicates *p* < 0.001, **** indicates *p* < 0.0001.

The tea samples used in this study were prepared from fresh leaves of the *Shuixian* cultivar grown in the same tea garden at Jiulongshan Tea Factory, Wuyixing Tea Industry Co., Ltd., Wuyishan, China (longitude 117.948425, latitude 27.711248). Fresh leaves were plucked according to the standard of one bud with three or four leaves. On 27 April 2025, dew-free fresh leaves were harvested under clear weather conditions, with a moisture content of 77.31%. The harvested leaves were processed into WRT using a traditional manufacturing procedure in a controlled processing room at 24 °C and 71% relative humidity by a tea master with more than 25 years of tea-processing experience (Figure 1A). The manufacturing process was performed with modifications based on the method described by Tan et al. [15], and consisted of withering, shaking and standing, fixation, rolling, and drying. Specifically, the fresh leaves were withered in a hot-air environment for 3.5 h, followed by shaking and standing for 9.5 h. The leaves were then subjected to fixation in a rotary drum at 270 °C for 5.0 min, rolled in a rolling machine for 5.5 min, and finally dried at 120 °C for 2.0 h. The tea samples processed from dew-free leaves were designated as DF.

To simulate dew conditions, controlled short-term leaf-wetting conditions were established. On the same day (27 April 2025), Shuixian tea plants from the same tea garden were subjected to continuous foliar water spraying for 1 h before plucking to ensure that each leaf was uniformly wetted before harvest. Dew-soaked leaves were harvested according to the same plucking standard, with a moisture content of 82.34%, and were processed into WRT using the same manufacturing procedure. The tea samples processed from dew-soaked leaves were designated as DS. No obvious differences were observed between the two groups of fresh leaves, except for their moisture contents. Three independent biological replicates were prepared for each group. All samples were stored at −20 °C in the dark until further analysis.

### 2.3. Sensory Evaluation and QDA of Tea Samples

Sensory evaluation was carried out in accordance with the Chinese National Standard GB/T 23776–2018 [16]. The appearance of each tea sample was first examined. Subsequently, 5 g of tea leaves were infused with 110 mL of boiling water in a standard tasting cup and brewed for 2, 3, and 5 min consecutively, followed by evaluation of aroma, liquor color, taste, and infused leaves. Quantitative descriptive analysis was performed with modifications to the method of [17]. Members of the Tea Industry Innovation Team at Fujian Agriculture and Forestry University participated in the sensory testing and were screened based on their ability to recognize aroma attributes and evaluate the flavor profile of WRT. The qualification criterion required an accuracy rate greater than 80% across all sensory tests. Ultimately, nine panelists were selected to form the sensory evaluation panel, comprising four males and five females, with a mean age of 33.44 ± 11.73 years and an age range of 22–60 years. Each aroma attribute was evaluated on a 10-point intensity scale (0 = not perceptible, 5 = moderate intensity, and 10 = extremely intense). Prior to formal evaluation, all panelists were trained using the established aroma descriptor system and unused WRT samples, covering five aroma attributes: floral aroma, fruity aroma, sweet aroma, woody aroma, and grassy off-odor. “Floral aroma” denotes blooming flowers; “fruity aroma” denotes ripe fruit; “sweet aroma” denotes honey; “woody aroma” denotes dry wood and smoked wood; and “grassy off-odor” denotes a mixed note of fresh grass, fatty, and fishy tones, representing a typical quality defect in tea processed from dew-soaked leaves. Training sessions were conducted two to three times per week, each lasting 1–2 h, until all panelists were proficient and consistent in using the aroma terminology and intensity scale. Each sample was evaluated in triplicate, and the final scores were calculated as the mean values for analysis. Tea falls within the domain of food research, and sensory evaluation does not require formal ethical approval; therefore, this study was not submitted to a human ethics committee for review. Nevertheless, the study adhered to relevant ethical standards for protecting participant rights and privacy. Specifically, all participants took part voluntarily; the study objectives, procedures, and potential risks were fully explained prior to sensory evaluation; experiments were conducted only after obtaining both written and verbal informed consent; personal information and data were kept strictly confidential and not disclosed without permission; and participants retained the right to withdraw from the study at any stage.

### 2.4. Quantitative and Qualitative Analysis of Volatile Compounds by HS-SPME/GC–MS

Volatile compounds (VOCs) in tea samples were extracted using a 65 μm polydimethylsiloxane/divinylbenzene (PDMS/DVB) fiber (Supelco, Bellefonte, PA, USA) through headspace solid-phase microextraction (HS–SPME). Briefly, 1.0 g of tea powder was placed in a 20 mL headspace vial, followed by the addition of 3 mL of boiling water and 10 μL of ethyl caprate internal standard solution (10 mg·L^−1^). The mixture was thoroughly homogenized, equilibrated at 60 °C for 15 min, extracted at the same temperature for 30 min, after which the SPME fiber was transferred to the GC–MS injector and desorbed for 5 min.

GC analysis was performed according to the laboratory-established method [9]. A Shimadzu GC-2030 gas chromatograph coupled with a QP2020 NX mass spectrometer (Shimadzu, Tokyo, Japan) was used for VOC identification. Separation was achieved on an SH-I-5Sil MS capillary column (30 m × 0.25 mm × 0.25 μm). High-purity helium (≥99.99%) was used as the carrier gas with a linear velocity of 48.40 cm/s. The injector temperature was set at 250 °C in split mode. The oven temperature program was as follows: initial temperature 35 °C (held for 2 min), ramped to 210 °C at 5 °C/min (held for 3 min), and then increased to 250 °C at 10 °C/min (held for 1 min).

MS conditions were as follows: ionization energy 70 eV, ion source and transfer line temperatures both set at 230 °C, full-scan mode with a mass range of *m*/*z* 35–350, and a solvent delay of 4 min. Qualitative analysis of VOCs was performed by comparing mass spectra and retention indices (RI) with those in the NIST database, combined with experimentally determined RI values using a C7–C40 n-alkane series under the same chromatographic conditions. Semiquantitative determination of VOCs was carried out using the internal standard method. Each sample was analyzed in triplicate. The Cvoc was calculated as follows:

(1) $$\mathrm{CVOC}=\frac{A_{\mathrm{VOC}}}{\mathrm{Astd}}\;\times \;\frac{m_{\mathrm{std}}}{m_{s}}$$

Note: C_voc_ represents the relative concentration of the VOC (μg/kg); A_voc_ represents the chromatographic peak area of the VOC; A_std_ represents the peak area of the internal standard; m_std_ represents the mass of the added internal standard; and ms represents the sample mass (g).

### 2.5. Calculation of rOAV and ACI

The relative odor activity value (rOAV) was determined by calculating the ratio of the concentration of each volatile compound to its odor threshold in the corresponding medium. Compounds with rOAV > 1 were generally considered key aroma-active compounds that contributed significantly to the overall aroma characteristics of the samples [18]. The aroma characteristic impact (ACI) was used to quantify the contribution of an individual component to the overall aroma profile [19]. Equations for rOAV and ACI are as follows:

(2) $$\mathrm{rOAV}\;=\;\frac{C_{i}}{\mathrm{OTi}}$$

(3) $$\mathrm{ACI}=\frac{\mathrm{rOAV}}{\sum_{k}\mathrm{rOAV}k}$$

Note: C_i_ represents the concentration of the VOC (μg/kg); OT_i_ represents the odor threshold of the VOC (μg/kg); and $\sum_{k}\mathrm{rOAV}k$ is the sum of rOAVs for all VOCs.

### 2.6. Analysis of Volatile Compounds Using GC-O

Olfactory determination of volatile compounds was performed using a Shimadzu ODE-2030 olfactometer (Shimadzu, Tokyo, Japan) connected to the GC–MS system, following the same extraction procedures and instrumental settings. Five assessors with the highest accuracy scores were selected to form the GC-O evaluation panel. A 0–10 scale was used to rate olfactory intensity, and compounds with scores ≥ 6 were defined as having significant sensory contribution [20]. The assessors recorded the aroma intensity and corresponding descriptors for each compound, and the mean aroma intensity values were calculated for subsequent analysis.

### 2.7. Absolute Quantification and Sensory Evaluation of Key Grassy Off-Odor Compounds in DS and DF Samples

According to the method described by [21], absolute quantification of four key grassy off-odor compounds, namely hexanal, heptanal, octanal, and (E,E)-2,4-heptadienal, was performed using external standard calibration curves. Standard solutions of the target compounds were mixed with ethyl caprate at concentration ratios of 1:5, 1:3, 1:1, 3:1, and 5:1. De-aromatized tea powder was used as the matrix to prepare five concentration levels of calibration samples. Following GC–MS analysis, calibration curves were established by plotting the concentration ratio of each target compound to ethyl caprate against the corresponding peak area ratio. The concentration ranges of all calibration curves covered the actual levels detected in tea samples and exhibited excellent linearity, with coefficients of determination (R2) exceeding 0.99 (Supplementary Table S1).

To verify the contributions of key grassy off-odor compounds, aroma addition experiments were performed. Briefly, 5 g of DS samples were placed in 110 mL standard tea tasting cups and served as the positive control, whereas DF samples were used as the negative control. Based on the absolute concentrations of the target compounds, the concentrations detected in DF samples were defined as low levels, while those detected in DS samples were defined as high levels. The experiment included both individual-compound additions and a mixture of all four compounds to evaluate the specific contribution of each compound to grassy off-odor perception. Prior to sensory evaluation, all samples were infused with boiling water for 2, 3, and 5 min. The grassy off-odor intensity of DF samples supplemented with the target compounds was then compared with that of DS samples. Sensory scores were assigned using a 10-point scale, with DF and DS defined as reference points corresponding to scores of 10 and 0, respectively. Each treatment was evaluated in triplicate to ensure the reliability and reproducibility of the results.

To further assess the relative contributions of individual grassy off-odor compounds, aroma recombination and omission experiments were conducted. Aroma recombination models were prepared according to the concentrations of hexanal, heptanal, octanal, and (E,E)-2,4-heptadienal detected in DS and DF samples (Supplementary Table S2). The low-concentration model contained hexanal (3.32 μg/mL), heptanal (1.32 μg/mL), octanal (4.07 μg/mL), and (E,E)-2,4-heptadienal (0.72 μg/mL), whereas the high-concentration model contained hexanal (7.84 μg/mL), heptanal (1.95 μg/mL), octanal (6.34 μg/mL), and (E,E)-2,4-heptadienal (5.80 μg/mL). A de-aromatized tea matrix (CK) was used as the negative control, while DS served as the positive control. Each aroma model was incorporated into the de-aromatized tea matrix at the corresponding concentrations. The de-aromatized tea matrix was prepared by repeatedly extracting DS samples with dichloromethane, followed by drying to remove endogenous aroma compounds. Prior to olfactory evaluation, the aroma samples were incubated in a 100 °C water bath for 2, 3, and 5 min. The similarity of grassy off-odor perception was subsequently evaluated by QDA using a 10-point intensity scale.

### 2.8. Molecular Docking to Predict the Binding Modes Between Key Grassy Off-Odor Compounds and Human Olfactory Receptors

Following a previous study [22], the SDF-format ligand files of four key volatile compounds—hexanal, heptanal, octanal, and (E,E)-2,4-heptadienal—were obtained from the PubChem database (https://pubchem.ncbi.nlm.nih.gov/), with PubChem CIDs of 6184, 8130, 454, and 5283321, respectively. Three-dimensional structural models of the olfactory receptor proteins were obtained from the UniProt database (Supplementary Table S3). Molecular docking was performed using AutoDock Vina 1.2.5, and the receptor proteins were prepared for docking by removing water molecules, adding polar hydrogen atoms, and assigning Kollman united-atom charges. The docking grid boxes were centered on the active sites of the receptor proteins and set to fully cover these sites. Three independent docking runs were performed. The optimal binding conformation was selected based on the lowest binding energy and the consistency of the docking poses across the independent runs. The simulation results were then further analyzed and visualized using Discovery Studio 2019.

### 2.9. Statistical Analysis

Statistical analyses were performed using IBM SPSS Statistics 23.0. The significance of differences was evaluated by one-way analysis of variance (ANOVA), with the significance level set at *p* < 0.05. Aroma analysis was conducted using the Metware Cloud platform (https://cloud.metware.cn/). Correlation network plots were constructed using Cytoscape 9.1, whereas violin plots, bar charts, and stacked plots were generated using GraphPad Prism 9.5.0. Radar charts and the aroma flavor wheel were drawn using Microsoft Excel 2019.

## 3. Results and Discussion

### 3.1. Sensory Evaluation and QDA of DS and DF

Sensory evaluation was conducted on DF and DS samples with respect to appearance, liquor color, taste, aroma, and infused leaves (Figure 1B, Supplementary Table S4) to reveal differences between the two. The results showed that DF exhibited tightly curled strips with a glossy and uniform appearance; its liquor was bright orange-yellow, the taste was mellow and brisk, the floral and fruity aromas were rich and persistent, and the infused leaves were plump with intact edges. In contrast, DS showed less tightness and gloss in the strips, with a less uniform appearance; its liquor was darker orange-yellow, the taste was thinner, the floral and fruity aromas were weaker with a distinct grassy off-odor, and the infused leaves displayed pronounced red edges and more broken margins. Overall, the sensory score of DS was significantly lower than that of DF.

To further investigate the influence of dew on the aroma characteristics of WRT, QDA was performed. The results indicated that the aroma acceptability score of DF (8.57) was significantly higher than that of DS (5.16) (Figure 1C). Aroma attribute analysis (Figure 1D) revealed distinct differences in flavor characteristics between the two samples. Regarding positive aroma attributes, DF exhibited more pronounced floral, fruity, sweet, and woody aromas, whereas DS displayed a prominent grassy off-odor, suggesting that the presence of dew exerted a negative impact on aroma quality. Previous research has demonstrated that acceptability and intensity are highly interdependent dimensions, and differences in acceptability among samples are typically attributed to variations in aroma intensity, familiarity, and harmony [23]. Evidently, DF achieved a more desirable state in terms of aroma intensity, familiarity, and balance, which enhanced the overall pleasantness perceived by assessors. In contrast, DS likely exhibited structural deficiencies in its volatile composition, resulting in lower acceptability scores due to internal disharmony.

Correlation analysis between aroma attributes and acceptability scores (Figure 1E) indicated that floral aroma, fruity aroma, sweet aroma, and woody aroma were significantly and positively associated with acceptability. These positive aroma attributes may act synergistically by co-activating partially overlapping olfactory receptors, thereby enhancing overall sensory pleasantness and improving consumer acceptability [24]. In contrast, grassy off-odor showed a significant negative correlation with acceptability (r = −0.97), suggesting a potential masking effect against positive aroma attributes. This phenomenon may be attributed to differences in odor quality and receptor activation patterns among aroma compounds. Overall, the presence of dew intensified the sensory perception of grassy off-odor, which in turn significantly reduced the overall aroma acceptability of WRT. To elucidate the chemical basis of grassy off-odor formation in DS, further analysis of the volatile composition is warranted.

### 3.2. Identification and Quantification of Aroma Compounds in DS and DF

**Figure 2:**
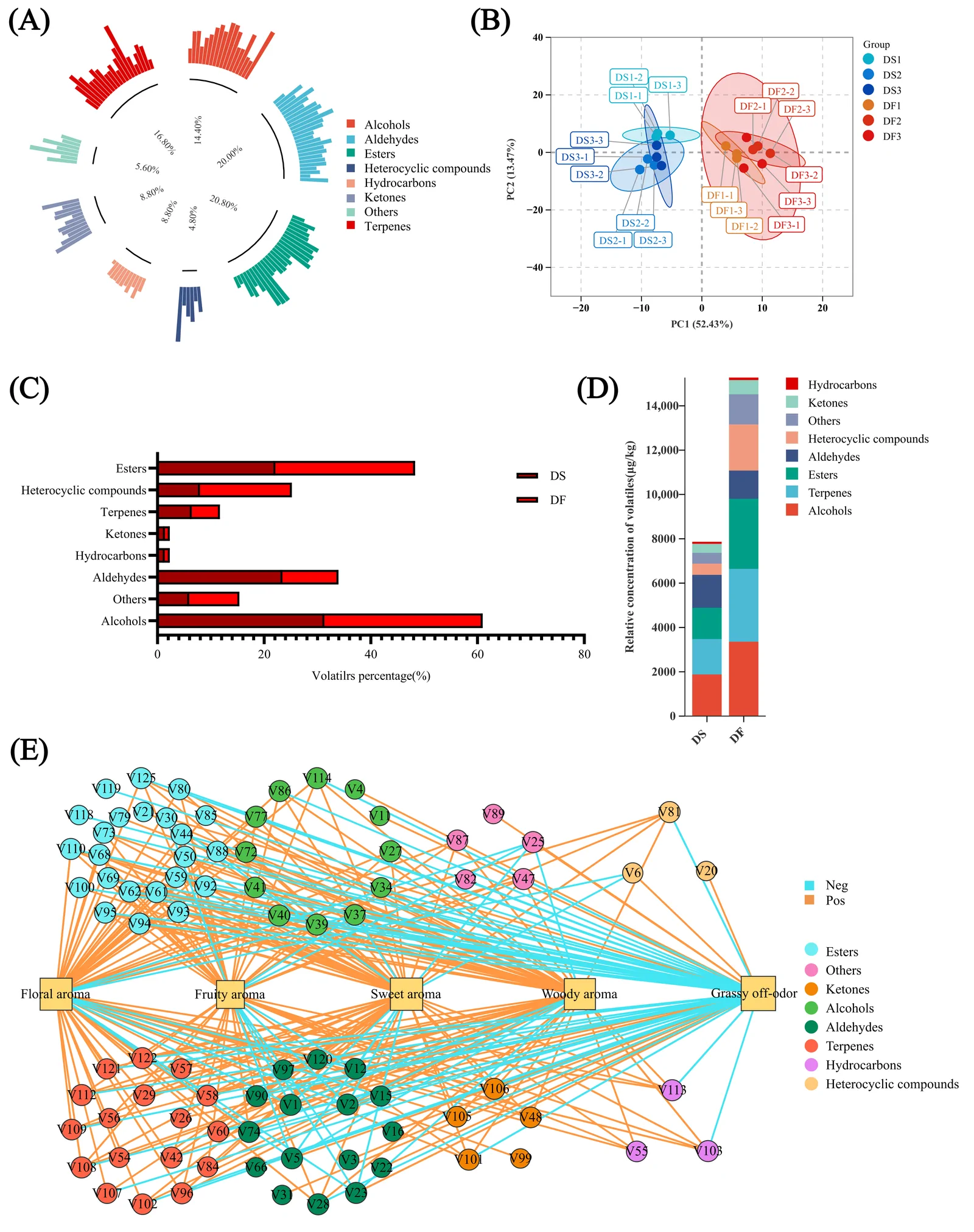
(**A**) Classification of 125 aroma components in DS and DF. (**B**) PCA plot. (**C**) Stacked plot of percentages of different aroma components. (**D**) Stacked plot of different aroma component contents. (**E**) Pearson correlation analysis between QDA score and aroma components, blue and orange indicate positive and negative correlations, respectively.

To elucidate the chemical basis of the grassy off-odor observed in DS, aroma compounds in DS and DF samples were analyzed using HS-SPME-GC–MS. A total of 125 aroma compounds were identified in the two samples (Figure 2A), which were classified into eight categories: esters (26 compounds, 20.80%), aldehydes (25 compounds, 20.00%), terpenes (21 compounds, 16.80%), alcohols (18 compounds, 14.40%), hydrocarbons (11 compounds, 8.80%), ketones (11 compounds, 8.80%), heterocyclic compounds (6 compounds, 4.80%), and others (7 compounds, 5.60%). Principal component analysis (PCA) results (Figure 2B) showed a clear separation between DS and DF samples based on their aroma compound compositions, with a cumulative contribution rate of 65.90%, indicating significant differences in aroma characteristics between the two groups.

The composition and structure of aroma compounds played an important role in determining the flavor characteristics of the samples. The results of aroma percentage and relative content (Figure 2C) showed that in DF, alcohols (23.40%) were the predominant compounds, followed by terpenes (21.55%) and esters (20.72%). Most alcohol compounds were closely associated with sweet and fruity aromas, ester compounds were related to floral, fruity, and sweet aromas, and terpenes typically contributed to floral and fruity characteristics [25]. The significantly higher contents of compounds associated with positive aroma attributes in DF likely enhanced its pleasant floral, fruity, sweet, and woody aroma characteristics. In DS, alcohols also dominated (25.28%), followed by terpenes (20.34%) and aldehydes (18.93%). Compared with DF, the relative abundance of aldehydes was markedly increased in DS. Previous studies have reported that most aldehydes are associated with off-odor attributes, such as grassy off-odor and dull notes, and their elevated levels may suppress the overall aroma quality [26]. Quantitative analysis further showed that the total content of aroma compounds in DF (15,273.44 μg/kg) was significantly higher than that in DS (7861.03 μg/kg) (Figure 2D). Previous research has indicated that deterioration in fresh leaf quality leads to a reduction in the total amount of aroma compounds in tea [6]. Compared with DF, the higher moisture content of DS fresh leaves was likely the main reason for its lower overall aroma compound concentration.

To further explore the influence of dew on the contents of compounds associated with different aroma attributes in DS and DF, Pearson correlation analysis was performed between QDA results and aroma compounds (r > 0.8, *p* < 0.05). The results (Figure 2E) showed that a total of 84 aroma compounds exhibited significant correlations with the five aroma attributes. As the characteristic aroma type of WRT, floral aroma was positively correlated with indole, geraniol, and phenylethyl alcohol. Indole, a key floral marker compound in oolong tea, plays a crucial role in flavor formation through activation of the terpene metabolic pathway [27], and its content in DF was significantly higher than that in DS. Fruity aroma was positively correlated with trans-nerolidol, linalool, and hexanoic acid, trans-2-hexenyl ester, while sweet aroma was positively correlated with geraniol, phenylethyl alcohol, and phenylacetaldehyde. Notably, both geraniol and phenylacetaldehyde were significantly higher in DF than in DS. Geraniol, a major contributor to rose-like and sweet notes in WRT, is closely related to enhanced geraniol synthase activity induced by leaf damage [28]. Phenylacetaldehyde imparts floral, caramel-like, and roasted notes and commonly exists in tea as a glycosidically bound form [29]. Additionally, woody aroma showed a significant positive correlation with trans-nerolidol, α-farnesene, and geranylacetone. Previous studies have reported that α-farnesene is an important woody and floral aroma compound in tea [30], and its content in DF was 2.32 times higher than that in DS. Overall, these compounds collectively contributed to the more intense and harmonious positive aroma characteristics of DF.

Grassy off-odor was positively correlated with hexanal, octanal, and (E,E)-2,4-heptadienal, totaling 70 associated compounds, most of which were aldehydes. These compounds were present at significantly higher levels in DS than in DF and imparted unpleasant odor characteristics such as grassy, fatty, earthy, and mushroom-like notes. Hexanal has been widely recognized as a marker compound of quality defects in tea, and its concentration directly reflects tea quality, serving as one of the main contributors to grassy off-odor [31]. Its content in DS was 2.46 times higher than that in DF. (E,E)-2,4-heptadienal, a characteristic compound associated with grassy and fatty notes in WRT, produces a pungent fishy or rancid odor when coexisting with 1-octen-3-ol (mushroom, earthy odor) [32]. Its content in DS was 2.70 times higher than that in DF. The interactions among these off-odor compounds may constitute the chemical basis for the grassy off-odor observed in DS. Complex interactive effects commonly occur among aroma compounds; when the levels of positive aroma compounds decrease, the sensory intensity of unpleasant ones is often amplified, thereby aggravating the overall unpleasant perception. These findings suggest that the presence of dew may affect the biosynthesis of aroma compounds in tea leaves, leading to the accumulation of aldehydes with undesirable odors, further intensifying the grassy off-odor, and consequently reducing the overall flavor quality of the tea [33].

### 3.3. Analysis of Aroma Compounds in DS and DF

**Figure 3:**
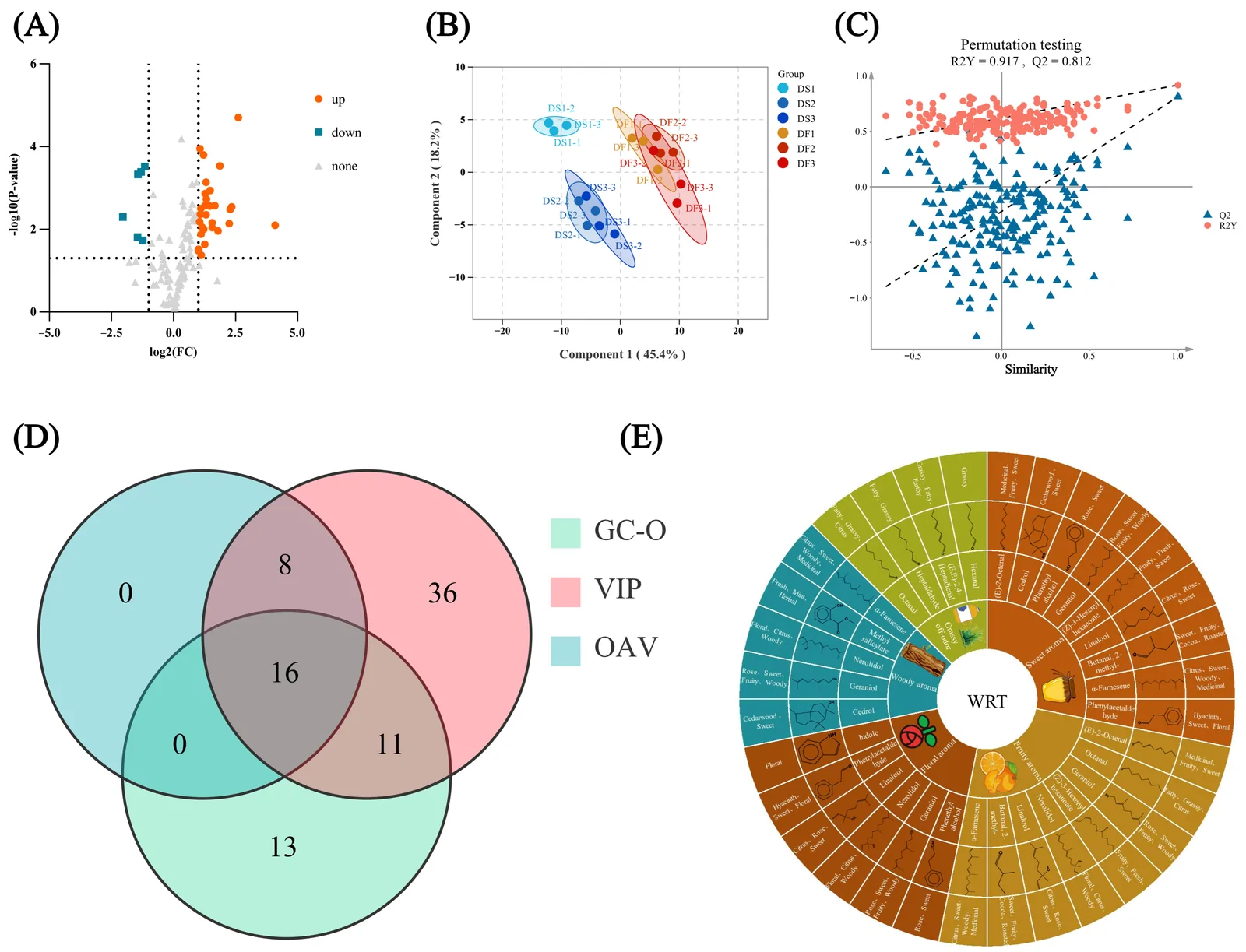
(**A**) Volcano plot of 125 aroma compounds in DS and DF. (**B**) OPLS–DA score plot for DS and DF (R^2^X = 0.956, R^2^Y = 0.998, and Q^2^ = 0.996). (**C**) Permutation testing of the OPLS-DA. (**D**) Venn diagram of VIP, rOAV and GC-O. (**E**) Aroma wheel of 16 major aroma active compounds in DS and DF.

A systematic analysis of the aroma compound compositions of the two WRT samples was conducted, and differential compounds were identified to elucidate the causes of aroma differences between DS and DF resulting from the presence of dew. A volcano plot was generated using *p* < 0.05 and |log2FC| > 0.5 as screening criteria. The results (Figure 3A) showed that, compared with DF, a total of 29 aroma compounds significantly decreased and 6 compounds significantly increased among the 125 identified compounds in DS (Supplementary Table S5). These compositional differences played an important role in shaping the flavor characteristics of the samples. Further orthogonal partial least squares discriminant analysis (OPLS-DA) was performed on the aroma compounds of DF and DS (Figure 3B). The results showed a clear discrimination between the two groups, with a cumulative explained variance of 63.60%, indicating significant differences in the aroma compound compositions of the two WRT samples. An OPLS–DA discriminant model was established based on differences in aroma compounds between the two groups of samples. A permutation test with 200 iterations was subsequently performed (R^2^ = 0.917 and Q^2^ = 0.812). As shown in Figure 3C, the intercept of the Q^2^ regression line on the y-axis was below zero, indicating that the model was not overfitted and exhibited high reliability.

The variable importance in projection (VIP) values reflected the contribution of each variable to the differentiation between groups, with VIP > 1.0 generally considered to indicate a significant contribution to sample discrimination. From the 125 identified aroma compounds in WRT, a total of 71 compounds were screened as significantly correlated with dew content (Supplementary Table S6). Further analysis revealed that these compounds exhibited marked differences in both content and aroma attributes between DS and DF samples. The 71 compounds with VIP > 1 included esters (21), aldehydes (13), alcohols (10), terpenes (12), ketones (4), heterocyclic compounds (3), hydrocarbons (4), and others (4). Compared with DS, 55 aroma compounds showed increased levels and 16 decreased levels in DF. The upregulated compounds were mainly esters, terpenes, and alcohols, while the downregulated ones were primarily aldehydes.

The aroma characteristics of tea represent a complex and integrated olfactory perception, arising from the combined stimulation of nasal olfactory receptors by diverse volatile compounds at different concentrations and proportions. This perception is jointly determined by the contents of aroma compounds and their corresponding odor thresholds. In total, 34 aroma compounds with calculable relative odor activity values were identified (Supplementary Table S7), of which 24 compounds exhibited rOAVs > 1. To further elucidate their contributions to different aroma attributes, detailed information, including odor thresholds in water, rOAV values, and odor characteristics, was listed in Table 1. The results showed that the top five aroma compounds ranked by aroma characteristic index accounted for cumulative relative contributions of 83.83% in DF and 86.85% in DS, respectively. In DF, the main contributors were linalool (rOAV: 1964.02; ACI: 55.23%), phenylacetonitrile (rOAV: 421.33; ACI: 11.85%), geraniol (rOAV: 251.73; ACI: 7.08%), indole (rOAV: 176.20; ACI: 4.95%), and octanal (rOAV: 167.91; ACI: 4.72%). Most of these compounds were associated with floral, fruity, and sweet aromas, contributing to pleasant positive aroma characteristics. In DS, the major contributors were linalool (rOAV: 1520.88; ACI: 54.86%), (E,E)-2,4-heptadienal (rOAV: 135.41; ACI: 13.16%), octanal (rOAV: 226.98; ACI: 8.19%), geraniol (rOAV: 179.60; ACI: 6.48%), and phenylacetonitrile (rOAV: 115.30; ACI: 4.16%). Among them, octanal and (E,E)-2,4-heptadienal contributed unpleasant grassy and fatty odors. Collectively, the significant differences in the composition of key aroma compounds between DS and DF constituted the main chemical basis for the observed differences in their aroma characteristics.

### 3.4. GC-O Analysis of Key Aroma Components in DS and DF

To further investigate the aroma attributes of volatile compounds, GC-O analysis was conducted on WRT samples processed from leaves with different dew contents. The GC-O results (Supplementary Table S8) showed that a total of 40 volatile aroma compounds were detected across all samples. These compounds exhibited a wide range of odor characteristics, including floral, fruity, sweet, woody, tobacco, fresh, grassy, fatty, mushroom-like, and fishy notes. In DF (Table 2), nine aroma compounds exhibited olfactory intensity scores ≥ 6: linalool (citrus, rose, sweet; intensity = 6.61), phenylacetaldehyde (hyacinth, sweet, fruity; intensity = 7.62), E-2-octenal (medicinal, fruity, sweet; intensity = 6.07), 2-acetyl-1H-pyrrole (roasted, sweet; intensity = 6.21), phenylethyl alcohol (rose, sweet; intensity = 6.38), hexyl isobutyrate (fruity, fresh; intensity = 6.43), (E,Z)-2,6-nonadienal (fresh, cucumber; intensity = 6.11), (E)-2-nonenal (cucumber, citrus, fresh, fatty; intensity = 6.34), and β-cyclocitral (woody, tobacco, citrus, floral; intensity = 6.19). These aroma compounds primarily contributed to the floral, fruity, sweet, and woody sensory characteristics of DF, playing a crucial role in shaping its positive aroma profile.

In the DS samples, ten volatile compounds exhibited olfactory intensity scores ≥ 6: heptanal (fatty, grassy; intensity = 6.05), octanal (fatty, grassy, soapy; intensity = 6.67), (E,E)-2,4-heptadienal (grassy, fatty, fishy; intensity = 7.38), phenylacetaldehyde (hyacinth, sweet, fruity; intensity = 6.90), E-2-octenal (medicinal, fruity, sweet; intensity = 6.45), 2-acetylpyrrole (roasted, sweet; intensity = 6.12), hexyl isobutyrate (fruity, fresh; intensity = 6.19), (E,Z)-2,6-nonadienal (fresh, cucumber; intensity = 6.62), (E)-2-nonenal (cucumber, citrus, fresh, fatty; intensity = 6.86), and β-cyclocitral (woody, tobacco, citrus, floral; intensity = 6.67). These volatile compounds collectively imparted grassy, fatty, fruity, and woody notes to the DS samples. Notably, the aldehydes octanal and (E,E)-2,4-heptadienal were perceived with strong intensities in DS, characterized by distinct grassy and fatty odors, indicating their major contributions to grassy off-odor formation. This observation was consistent with the traditionally recognized sensory characteristics of teas processed from dew-soaked leaves, which are typically described as grassy and dull. Overall, the GC–O results further confirmed that the presence of dew significantly altered the composition and balance of aroma compounds, representing a key factor responsible for the flavor differences between DS and DF.

### 3.5. Analysis of Key Aroma Compounds in DS and DF

Based on the OPLS-DA results (VIP > 1), odor activity values (rOAV > 1), and GC-O olfactory intensity data, key aroma compounds in WRT samples were comprehensively screened. A total of 16 aroma compounds were identified as key contributors to the overall aroma characteristics of the samples (Figure 3D). An aroma wheel of WRT was constructed using the odor attributes of these compounds to visualize the sensory characteristics of DS and DF (Figure 3E). In DF, aroma compounds such as indole, phenylacetaldehyde, α-farnesene, linalool, methyl salicylate, trans-nerolidol, cis-3-hexenyl hexanoate, geraniol, phenylethyl alcohol, and cedrol exhibited higher sensory intensities than those in DS and were perceptible to the human olfactory system. The interactions among these compounds may collectively enhance the distinctive floral aroma, fruity aroma, sweet aroma, and woody aroma characteristics of DF. In DS, compounds including (E,E)-2,4-heptadienal, E-2-octenal, octanal, hexanal, 2-methylbutanal, and heptanal exhibited higher sensory intensities than in DF, and these compounds were likely associated with the grassy, fatty, and dull odor characteristics unique to DS.

**Figure 4:**
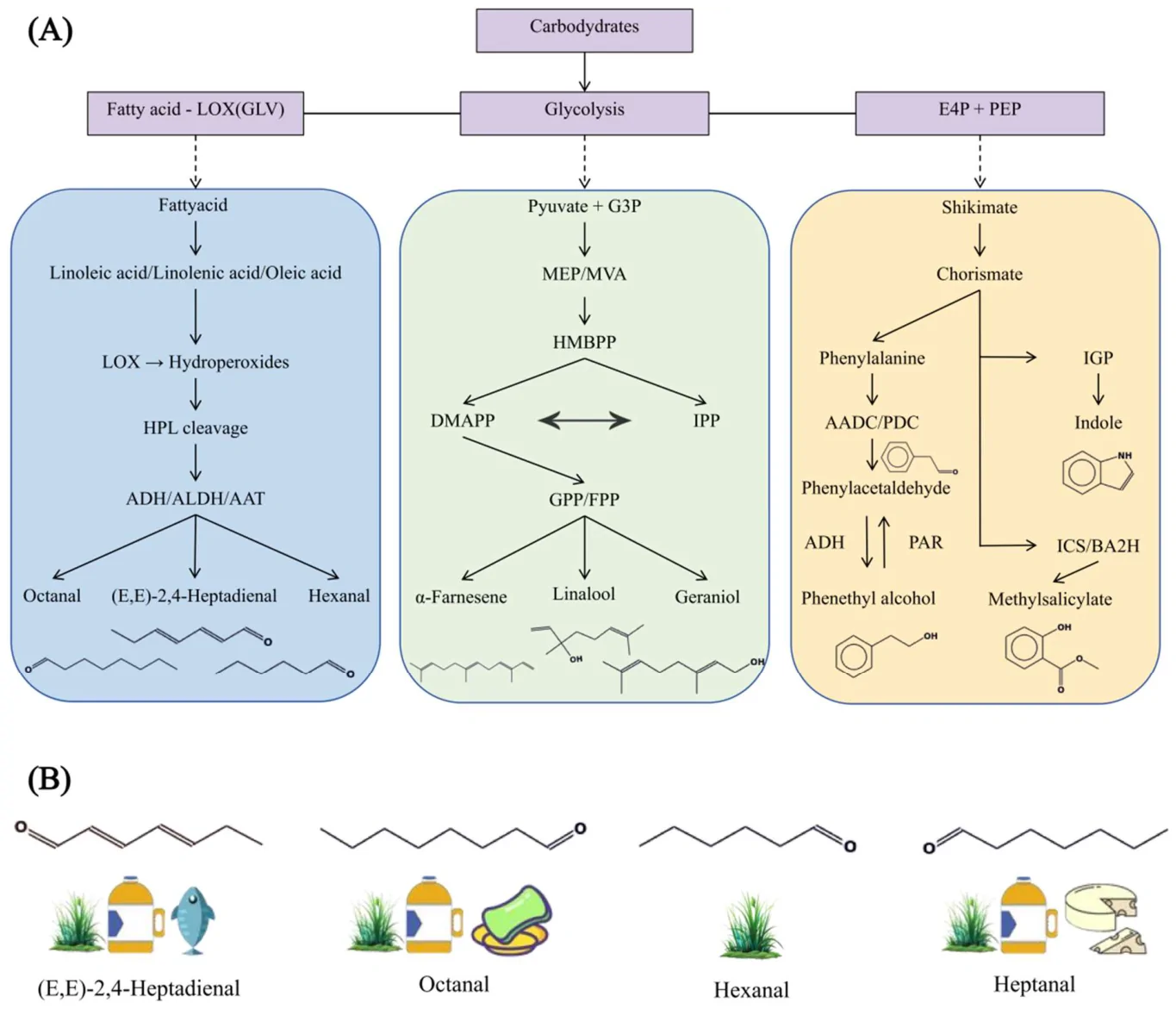
(**A**) Biosynthetic pathway of 16 key aroma compounds in WRT. LOX, lipoxygenase; HPL, hydroperoxide lyase; ADH, alcohol dehydrogenase; ALDH, aldehyde dehydrogenase; AAT, alcohol acyltransferase; G3P, glyceraldehyde 3-phosphate; MEP, 2-C-methyl-D-erythritol 4-phosphate pathway; MVA, mevalonate pathway; HMBPP, (E)-4-hydroxy-3-methylbut-2-enyl diphosphate; DMAPP, dimethylallyl diphosphate; IPP, isopentenyl diphosphate; GPP, geranyl diphosphate; FPP, farnesyl diphosphate; E4P, erythrose 4-phosphate; PEP, phosphoenolpyruvate; AADC, aromatic amino acid decarboxylase; PDC, phenylpyruvic acid decarboxylase; PAR, phenylacetaldehyde reductase; IGP, indole-3-glycerol phosphate; ICS, isochorismate synthase; BA2H, benzoic acid 2-hydroxylase. (**B**) Molecular structure and odor of (E,E)-2,4-heptadienal, Octanal, Hexanal and Heptanal.

To elucidate the influence of dew presence on the formation of aroma compounds in WRT, the biosynthetic pathways of 16 key aroma compounds were summarized, and the formation mechanisms of different aroma types were interpreted from a metabolic perspective (Figure 4A). Linalool and geraniol, the primary floral aroma compounds in WRT, were mainly derived from the methylerythritol phosphate (MEP) pathway [35]. As structural isomers, they can be interconverted, and their synthesis is regulated by both enzymatic activity and environmental factors, such as temperature, humidity, and oxygen availability. α-Farnesene, nerolidol, and cedrol share the same precursor, farnesyl pyrophosphate (FPP), and are synthesized through different enzymatic catalyses, a process typically associated with the release of defense-related volatiles triggered by plant stress or tissue damage [36]. Indole and methyl salicylate are synthesized via the shikimate pathway. The synthesis of indole is highly dependent on continuous mechanical stress during the “tossing” stage, which provides essential enzymatic activation signals for indole formation [27]. Phenylacetaldehyde and phenylethyl alcohol are also produced through the shikimate pathway, originating from phenylalanine via a series of enzymatic reactions involving transaminases and decarboxylases. Phenylacetaldehyde, a representative compound responsible for the sweet floral notes of Oriental Beauty tea, accumulates under dual regulation induced by tea leafhopper puncturing—physical damage directly releases precursors and simultaneously upregulates the expression of related enzyme genes—and this process is closely associated with leaf moisture content [37]. Furthermore, the interconversion between phenylacetaldehyde and phenylethyl alcohol is precisely regulated by redox enzymes such as ADH and PAR, maintaining the dynamic balance of aroma composition. Previous studies have confirmed that aldehydes such as hexanal, (E,E)-2,4-heptadienal, and octanal are typical markers of lipid oxidation, mainly derived from the degradation of unsaturated fatty acids. During tea processing, linoleic and α-linolenic acids are oxidized by lipoxygenase (LOX) to form hydroperoxides, which are subsequently cleaved by hydroperoxide lyase (HPL) to produce specific aldehydes [38]. Specifically, the 13-hydroperoxide of linoleic acid generates hexanal, while that of α-linolenic acid produces (E,E)-2,4-heptadienal; octanal and heptanal can also be formed through similar LOX/HPL-mediated pathways [39]. The concentrations of these aldehydes are generally negatively correlated with tea quality and can serve as potential indicators of raw material defects or inappropriate processing [40]. This finding is consistent with the grassy off-odor and dull sensory characteristics observed in DS.

These findings suggest that simulated dew treatment may inhibit the biosynthesis of certain aroma compounds while promoting lipid peroxidation and other secondary metabolic pathways, thereby reducing the aroma quality of the finished tea [33]. On the one hand, the biosynthesis of positive aroma compounds, such as linalool, indole, and phenylacetaldehyde, may be inhibited. On the other hand, lipid peroxidation in tea leaves may be accelerated under dew-soaked conditions, resulting in the substantial accumulation of aldehydes with grassy odors, including hexanal, (E,E)-2,4-heptadienal, and octanal. Collectively, these changes contribute to the distinctive “grassy” or “dull” notes in the tea aroma [41]. Huang et al. [42] similarly reported that the accumulation of aldehydes, including hexanal, (E)-2-hexenal, and (E,E)-2,4-heptadienal, was significantly affected by preharvest rainfall stress, which could readily accelerate lipid peroxidation in tea leaves, consistent with the findings of the present study. Further correlation analysis showed that hexanal (r = 0.97), heptanal (r = 0.93), octanal (r = 0.88), and (E,E)-2,4-heptadienal (r = 0.98) were significantly positively correlated with the grassy off-odor scores, suggesting that these compounds may be key contributors to the characteristic grassy off-odor of DS. The molecular structures and aroma attributes of the four compounds are shown in Figure 4B.

### 3.6. Validation of Key Grassy Off-Odor Compounds in DS via Sensory Evaluation

**Figure 5:**
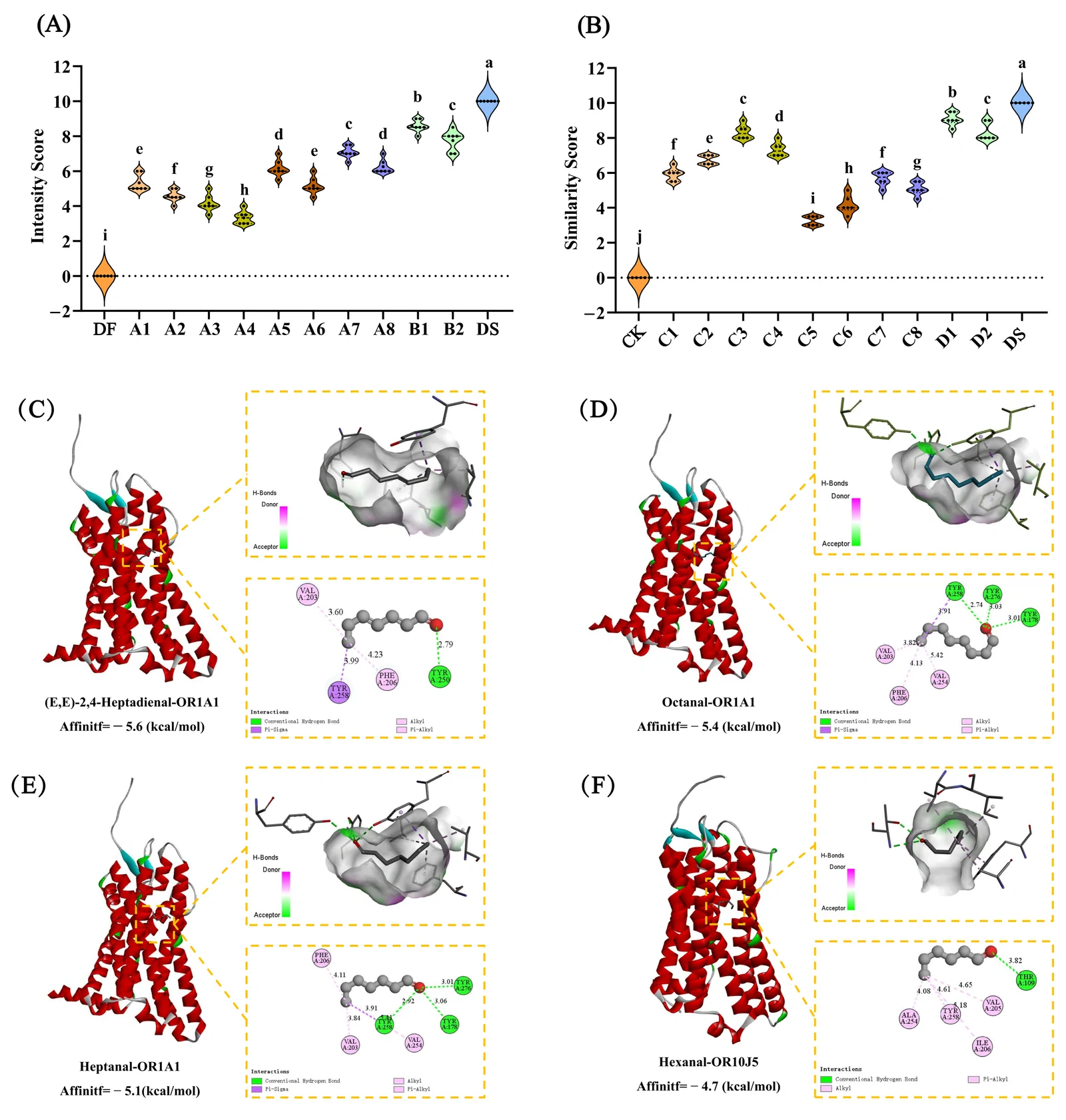
(**A**) Intensity scores of the grassy off-odor addition simulation test. (**B**) Similarity scores of the grassy odor reconstitution and omission simulation tests. Different letters indicate significant differences when *p* < 0.05. (**C**) Molecular docking interaction diagram between (E,E)-2,4-heptadienal and olfactory receptor OR1A1. (**D**) Molecular docking interaction diagram between octanal and olfactory receptor OR1A1. (**E**) Molecular docking interaction diagram between heptanal and olfactory receptor OR1A1. (**F**) Molecular docking interaction diagram between hexanal and olfactory receptor OR10J5.

To further validate the contributions of the selected compounds to grassy off-odor, a human sensory evaluation was conducted to assess their roles in eliciting off-odor perception. The DS sample served as the positive control, while the DF sample was used as the negative control. As shown in Figure 5A, the grassy off-odor score of DF increased with the addition of grassy off-odor compounds. Among the four compounds, DF exhibited the highest grassy off-odor score upon the addition of (E,E)-2,4-heptadienal (high concentration: 7.08; low concentration: 6.25), followed by octanal (high concentration: 6.17; low concentration: 5.17), hexanal (high concentration: 5.33; low concentration: 4.58), and heptanal (high concentration: 4.17; low concentration: 3.33). When all four compounds were added simultaneously, the grassy off-odor score of the mixture increased significantly (high concentration: 8.58; low concentration: 7.75), indicating an additive effect whereby the combined components elicited a stronger off-odor response than any single compound alone. This enhancement may also arise from synergistic interactions among the compounds. Among the four grassy off-odor compounds, (E,E)-2,4-heptadienal and octanal caused the greatest reduction in acceptability of DF, characterized by intense grassy, fatty, and fishy odors, suggesting that these two were the core contributors to the grassy off-odor. Hexanal and heptanal, which imparted grassy and fatty notes, appeared to play auxiliary roles by enhancing the overall unpleasant perception through interactions with other grassy off-odor compounds.

To further examine the relative contributions of the four aroma compounds to the unpleasant grassy off-odor attribute, omission tests were performed to systematically evaluate their roles in sensory formation. The results (Figure 5B) showed significant differences in the contributions of the four compounds to the grassy off-odor. Removal of (E,E)-2,4-heptadienal resulted in a significant decrease in similarity scores, followed by octanal and hexanal, whereas removal of heptanal caused the least change. Notably, in both high- and low-concentration gradients, the trend in similarity scores remained consistent, and the relative contribution order of the compounds did not change significantly. This indicated that the interaction pattern of these compounds was relatively stable and less affected by concentration variations. Such a phenomenon might be attributed to the olfactory saturation effect for mixed odors, where the relative intensity relationships among components tend to remain constant within a certain concentration range, ensuring the stability and reproducibility of sensory contribution patterns. From the perspective of aroma attributes, (E,E)-2,4-heptadienal and octanal exhibited strong grassy, fatty, and soapy notes that markedly intensified the grassy off-odor characteristic [43]. This may be attributed to their pungent odor properties and their significant negative correlation with tea quality grade [20]. Hexanal showed a single grassy note with lower irritation, indicating a relatively limited contribution to the overall grassy off-odor. Heptanal, described as having an “unpleasant pungent fatty odor” at higher concentrations but a faint fruity note at lower concentrations, may contribute less to the overall unpleasant perception of grassy off-odor [44].

### 3.7. Elucidation of the Binding Modes Between Key Grassy Off-Odor Compounds and Human Olfactory Receptors Based on Molecular Docking

Four key volatile compounds responsible for the aroma quality differences in WRT processed from dew-soaked leaves were identified, and their metabolic pathways during processing were elucidated. To characterize the binding modes between small-molecule compounds and olfactory receptors and to explore the perceptual mechanism of grassy off-odor in humans, molecular docking analysis was performed. Based on previous studies, twelve widely studied olfactory receptors (OR5K1, OR7A5, OR8H1, OR11H7, OR2M3, OR3A1, OR1A1, OR2W1, OR2J2, OR6A2, OR10A6, and OR10J5) were selected for binding conformation analysis [45]. Among them, OR6A2, a class II olfactory receptor, has been identified as selectively recognizing medium-chain aldehydes [46], whereas OR1A1, OR2W1, and OR10A6 have been reported to respond to various aliphatic aldehydes, including heptanal and octanal [47,48,49]. The results showed that the binding energies between different olfactory receptors and the key volatile compounds varied from −5.6 to −3.6 kcal/mol (Supplementary Table S9, Figure 5C–F). The average binding energies of the four key volatile compounds with the 12 olfactory receptors were as follows: (E,E)-2,4-heptadienal, −4.78 kcal/mol; octanal, −4.72 kcal/mol; heptanal, −4.43 kcal/mol; and hexanal, −4.14 kcal/mol. All binding energies were below zero, indicating that the intermolecular interactions were spontaneous [50]. Among the tested olfactory receptors, (E,E)-2,4-heptadienal, octanal, and heptanal exhibited the strongest binding affinities for OR1A1, with binding energies of −5.6, −5.4, and −5.1 kcal/mol, respectively, whereas hexanal exhibited the strongest binding affinity for OR10J5 (−4.7 kcal/mol).

In OR1A1, (E,E)-2,4-heptadienal formed a conventional hydrogen bond with Tyr250, while hydrophobic interactions were observed with residues such as Tyr258, Val203, and Phe206, which jointly stabilized its binding conformation. The conjugated double bonds in its molecular structure facilitated stable interactions with hydrophobic residues [51]. In OR1A1, octanal formed conventional hydrogen bonds with several tyrosine residues, including Tyr178, Tyr258, and Tyr276, through its aldehyde oxygen atom, and the stability of the complex was further enhanced by hydrophobic interactions with Val203, Val254, and Phe206. Heptanal formed a hydrogen-bonding network with Tyr178, Tyr258, and Tyr276 in OR1A1, and bound to Val203, Val254, and Phe206 through hydrophobic interactions. Compared with octanal, heptanal showed slightly lower binding affinity, which may be related to its shorter carbon chain and the resulting weaker contribution of hydrophobic interactions. Extensive hydrophobic interactions contribute to the stability of the complex and enhance the intensity of olfactory stimulation [52]. In OR10J5, hexanal was mainly stabilized by dual hydrogen-bonding interactions with Thr109, and bound to Ala254, Val205, and Ile206 through hydrophobic interactions.

Overall, molecular docking provided preliminary support for the GC–O findings. The presence of hydrophobic interactions and hydrogen bonds helped explain the binding affinities between these aroma compounds and their olfactory targets, providing molecular-level insights into the human olfactory perception of the key compounds responsible for the grassy off-odor in DS. However, the limitations of computational simulations arising from the complexity of biological systems must be acknowledged. The perceptual effects of these key odor-active compounds were evaluated solely through human sensory assessment. Given the complexity of aroma systems, future studies should further investigate the interactive effects among aroma compounds with different sensory attributes.

## 4. Conclusions

This study systematically investigated the key aroma-active compounds responsible for the grassy off-odor in WRT processed from dew-soaked leaves by integrating GC–MS, GC–O, and quantitative descriptive analysis, and a corresponding aroma wheel was constructed to visualize its sensory characteristics. A total of 16 key aroma compounds were identified, among which four compounds showed significant positive correlations with grassy off-odor perception. Metabolic network analysis indicated that dew treatment was closely associated with changes in the aroma composition of WRT. These changes may be associated with accelerated lipid peroxidation in tea leaves in the presence of dew, which could affect central metabolic processes and consequently influence WRT aroma formation. Aroma addition and omission tests confirmed that (E,E)-2,4-heptadienal, octanal, hexanal, and heptanal were the major contributors to the grassy off-odor, with (E,E)-2,4-heptadienal making the greatest contribution. Molecular docking analysis further indicated stable interactions between the grassy off-odor compounds and the key olfactory targets OR1A1 and OR10J5. These results provided molecular-level support for the GC–O identifications and a theoretical basis for understanding the potential mechanisms underlying human olfactory perception of the grassy off-odor.

Overall, this study provides new insights into the chemical basis and potential perceptual mechanisms of grassy off-odor formation in WRT processed from dew-soaked leaves. It also provides a theoretical basis for optimizing fresh-leaf processing under rainy conditions and improving the quality consistency of WRT. Although this study clarified the effect of the presence of dew on aroma quality, it did not fully reproduce the interactions among multiple microenvironmental factors during natural dew formation under specific conditions. The formation of these grassy off-odor compounds requires further verification through analyses of free fatty acids, lipid oxidation products, and lipoxygenase activity. Therefore, future studies may use controlled growth environments to simulate natural dew conditions and investigate the interactions among factors such as different tea plant cultivars, harvesting times, and field variations, thereby systematically elucidating the interactions among environmental factors and their potential roles in tea quality formation.

## Figures and Tables

**Table 1:** 24 key aroma compounds with rOAV > 1 in DS and DF.

Volatile Compounds	Odor Descriptors ^a^	Odor Threshold in Water ^b^ (μg/kg)	DS		DF	
			rOAV	ACI	rOAV	ACI
Indole	Floral	11	28.83	1.04%	176.20	4.95%
Phenylacetaldehyde	Hyacinth, Sweet, Floral	5.2	62.33	2.25%	77.77	2.19%
Benzeneacetonitrile	Aromatic	2.04	115.30	4.16%	421.33	11.85%
*α*-Farnesene	Citrus, Sweet, Woody, Medicinal	87	11.71	0.42%	27.10	0.76%
Hexanal	Grassy	2.5	89.78	3.24%	36.46	1.03%
(*E,E*)-2,4-Heptadienal	Grassy, Fatty, Fishy, Earthy	0.32	364.92	13.16%	135.41	3.81%
Butanoic acid, 2-methyl-, hexyl ester	Fruity, Sweet	22	5.43	0.20%	13.49	0.38%
2-Methylbutanal	Sweet, Fruity, Cocoa, Roasted	1.2	21.04	0.76%	11.98	0.34%
Linalool	Citrus, Rose, Sweet	0.22	1520.88	54.86%	1964.02	55.23%
Methyl salicylate	Fresh, Minty, Herbal	40	2.37	0.09%	3.87	0.11%
Nerolidol	Floral, Citrus, Woody	250	1.12	0.04%	5.66	0.16%
(*Z*)-3-Hexenyl hexanoate	Fruity, Fresh, Sweet	16	16.73	0.60%	41.49	1.17%
Benzene, (2-nitroethyl)-	Floral, Woody	2	20.29	0.73%	96.51	2.71%
Gamma-Nonanolactone	Creamy, Sweet, Coconut	9.7	0.62	0.02%	1.07	0.03%
Geraniol	Rose, Sweet, Fruity, Woody	1.1	179.60	6.48%	251.73	7.08%
Phenethyl alcohol	Rose, Sweet	140	0.76	0.03%	1.63	0.05%
Heptanal	Fatty, Grassy, Citrus	3	18.59	0.67%	12.61	0.35%
3-Methylbutyraldehyde	Fruity, Sweet, Apple	4.4	1.91	0.07%	0.70	0.02%
(*E*)-2-Heptenal	Cucumber, Grassy, Fatty	17	1.44	0.05%	0.78	0.02%
Benzyl alcohol	Sweet, Fruity, Floral	100	1.19	0.04%	1.51	0.04%
Cedrol	Cedarwood, Sweet	0.5	36.60	1.32%	73.60	2.07%
Octanal	Fatty, Grassy, Soapy, Citrus	0.587	226.98	8.19%	167.91	4.72%
(*E*)-2-Octenal	Medicinal, Fruity, Sweet	3	27.51	0.99%	20.73	0.58%
2-Pentylfuran	Fruity	5.8	16.42	0.59%	12.80	0.36%

^a^ The odor description eferred to the database (The Good Scents Company Information System, http://www.thegoodscentscompany.com/ (accessed on 12 July 2026)). ^b^ The odor thresholds were obtained from literature (Van Gemert, 2011 [34]).

**Table 2:** 12 aroma compounds in DS and DF with aroma olfactory Score > 6.

No.	#CAS	Volatile Compounds	Odor Descriptors ^a^	Aroma Intensity	
				DS	DF
V12	111-71-7	Heptanal	Fatty, Grassy, Citrus	6.05	4.05
V22	124-13-0	Octanal	Fatty, Grassy, Soapy, Citrus	6.67	5.00
V23	4313-03-5	(*E*,*E*)-2,4-Heptadienal	Grassy, Fatty, Fishy, Earthy	7.38	5.78
V28	122-78-1	Phenylacetaldehyde	Hyacinth, Sweet, Fruity	6.90	7.62
V31	2548-87-0	(*E*)-2-Octenal	Medicinal, Fruity, Sweet	6.45	6.07
V32	1072-83-9	2-Acetyl-1H-pyrrole	Roasted, Sweet	6.12	6.21
V39	78-70-6	Linalool	Citrus, Rose, Sweet	5.94	6.61
V41	60-12-8	Phenethyl alcohol	Rose, Sweet	5.74	6.38
V50	2349-07-7	Hexyl isobutyrate	Fruity, Fresh	6.19	6.43
V52	557-48-2	(2*E*,6*Z*)-Nona-2,6-dienal	Cucumber, Fresh, Sweet	6.62	6.11
V53	18829-56-6	(*E*)-2-Nonenal	Cucumber, Citrus, Fresh, Fatty	6.86	6.34
V66	432-25-7	*β*-Cyclocitral	Woody, Tobacco, Rose, Sweet	6.67	6.19

^a^ The odor description referred to the database (The Good Scents Company Information System, http://www.thegoodscentscompany.com/).

## Data Availability

The original contributions presented in this study are included in the article/Supplementary Materials. Further inquiries can be directed to the corresponding authors.

## Supplementary Materials

The following supporting information can be downloaded at https://www.mdpi.com/article/10.3390/foods15162894/s1, Table S1: Grassy off-odor addition tests; Table S2: Grassy off-odor recombination and omission tests; Table S3: Key information on 12 human olfactory receptors associated with grassy off-odor; Table S4: Sensory evaluation of DS and DF; Table S5: Volatile component substance information of DS and DF; Table S6: 71 key aroma components with VIP > 1 in the DS and DF; Table S7: rOAV of volatile compounds among different aroma types of DS and DF; Table S8: Aroma olfactometry scores for DS and DF; Table S9: Binding energy scores of four key aroma-active compounds in DS with 12 olfactory receptors.
